# HDAC1 controls *CIP2A* transcription in human colorectal cancer cells

**DOI:** 10.18632/oncotarget.8406

**Published:** 2016-03-26

**Authors:** Manjola Balliu, Cristina Cellai, Matteo Lulli, Anna Laurenzana, Eugenio Torre, Alessandro Maria Vannucchi, Francesco Paoletti

**Affiliations:** ^1^ Department of Experimental and Clinical Medicine, University of Florence, 50134 Firenze, Italy; ^2^ Department of Biomedical, Experimental and Clinical Sciences “Mario Serio”, 50134 Firenze, Italy

**Keywords:** HDAC-inhibitor, HDAC1, CIP2A transcription, PP2A, CRC cells

## Abstract

This work describes the effectiveness of HDAC-inhibitor (S)-2 towards colorectal cancer (CRC) HCT116 cells *in vitro* by inducing cell cycle arrest and apoptosis, and *in vivo* by contrasting tumour growth in mice xenografts. Among the multifaceted drug-induced events described herein, an interesting link has emerged between the oncoprotein histone deacetylase HDAC1 and the oncogenic Cancerous Inhibitor of Protein Phosphatase 2A (CIP2A) which is overexpressed in several cancers including CRCs. HDAC1 inhibition by (S)-2 or specific siRNAs downregulates *CIP2A* transcription in three different CRC cell lines, thus restoring the oncosuppressor phosphatase PP2A activity that is reduced in most cancers. Once re-activated, PP2A dephosphorylates pGSK-3β(ser9) which phosphorylates β-catenin that remains within the cytosol where it undergoes degradation. The decreased amount/activity of β-catenin transcription factor prompts cell growth arrest by diminishing *c-Myc* and *cyclin D1* expression and abrogating the prosurvival Wnt/β-catenin signaling pathway. These results are the first evidence that the inhibition of HDAC1 by (S)-2 downregulates *CIP2A* transcription and unleashes PP2A activity, thus inducing growth arrest and apoptosis in CRC cells.

## INTRODUCTION

Epigenetic changes are reversible rearrangements of chromatin capable of modulating gene expression in the cell without altering DNA sequence. Acetylation is the most widely studied post-translational modification of histones [1] and results from the balanced activity of two families of enzymes, namely the histone acetyltransferases (HATs) and histone deacetylases (HDACs) catalyzing acetylation/deacetylation of histones, respectively, and thereby providing DNA accessibility to transcription factors [2–5].

In cancer cells HDACs are typically overexpressed [6–9] and represent, therefore, the targets for diverse natural/synthetic compounds acting as HDAC-inhibitors (HDACis) [10–12]. Some of them have successfully been used in the clinic, either alone or in combination with conventional cytotoxics, as potent antineoplastic drugs to support current therapy [10, 11, 13]. Indeed, HDACis have proven effective in promoting cell cycle arrest and apoptosis in various types of tumours through the generation of reactive oxygen species, activation of caspase cascade, disruption of mitochondrial integrity, increase in autophagy [10], and suppression of pro-survival pathways [14, 15]. These events were at least in part mediated by acetylation of histones and nonhistone key regulatory proteins [16] including p53, GATA1, GATA2, retinoic acid receptor, NF-kB and cytoskeletal proteins like α-tubulin [17–19].

Previously, we described a new series of HDACis generated through the hybridization of 1,4-benzodiazepine (BDZ) ring with SAHA or oxamflatin [20] yielding compounds that displayed powerful anticancer properties against human malignant cells of acute myeloid leukemia [21], prostate adenocarcinoma [22], metastatic melanoma [15] and, notably, were safe to normal mice *in vivo* up to high dosages [15, 21].

The study herein aimed at evaluating the effectiveness of a specific HDACi, termed (S)-2, towards human colorectal cancer (CRC) cells HCT116 (and partly also HT-29 and HCT8) *in vitro* and at describing mechanisms underlying drug-induced cell growth arrest and apoptosis. Moreover, the drug showed to be also effective *in vivo* by contrasting HCT116 cell growth in mice xenografts.

Our results point to a crucial involvement of serine/threonine phosphatases and, in particular, of their physiological inhibitors, as mediators of anticancer properties of (S)-2 in CRC cells. Furthermore, these findings disclose a new role for HDAC1 in governing transcription of the oncogenic *Cancerous Inhibitor of Protein Phosphatase 2A (CIP2A)* that is known to be overexpressed in numerous cancers [23, 24] including CRCs [25]. To our knowledge, such a molecular link between HDAC1 and CIP2A has not been reported previously and may help, therefore, to understand the widespread anticancer effectiveness of several HDACis, including (S)-2, that recognize HDAC1 as a specific target.

## RESULTS

### (S)-2 prompts growth arrest and apoptosis in HCT116 colorectal carcinoma cells

**Figure 1:**
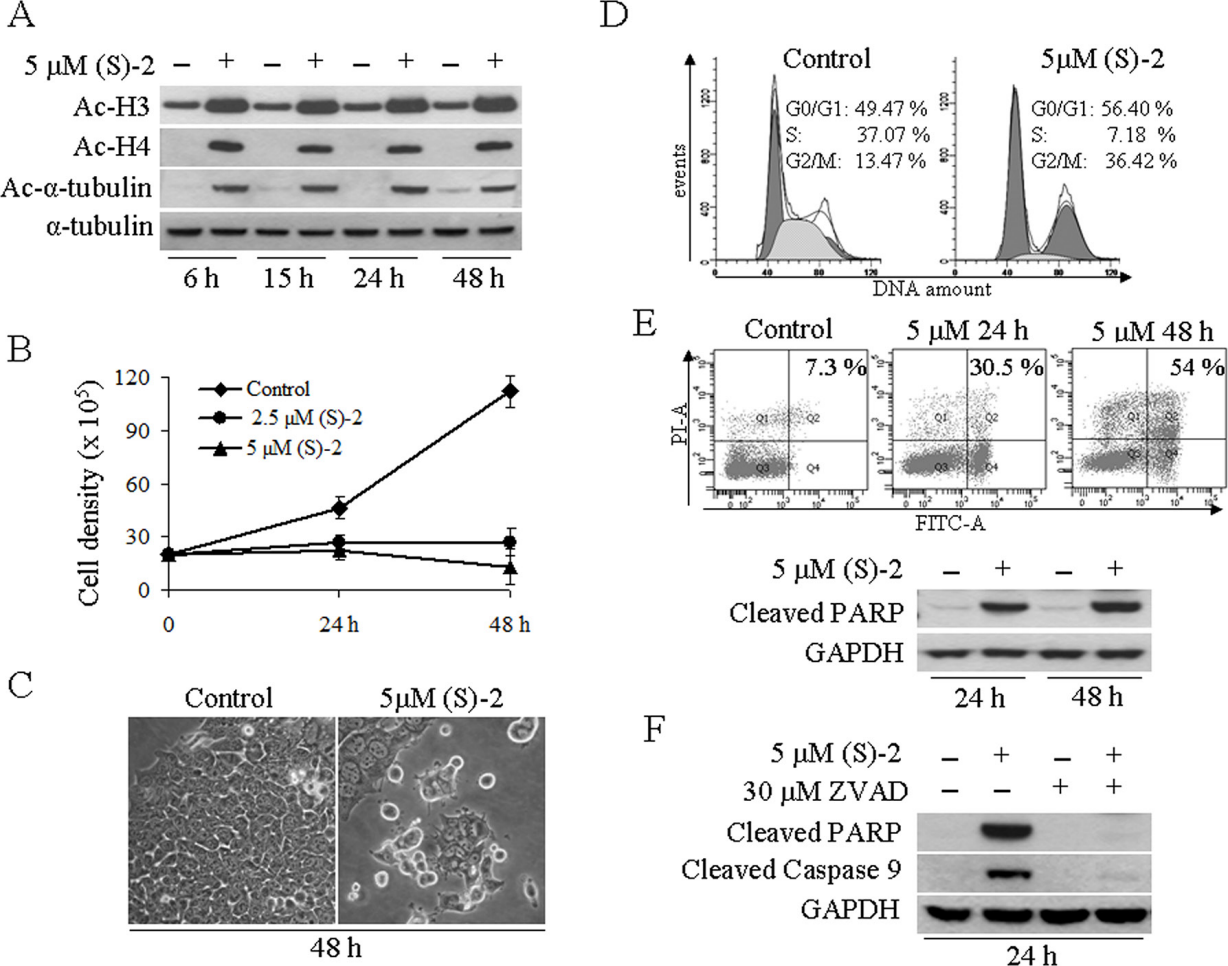
(S)-2 induced growth arrest and apoptosis in HCT116 cells (**A**) HCT116 cells were incubated for 6, 15, 24 and 48 h without/with 5 μM (S)-2 and then processed by Western blot and immunostained for the acetylated histone H3/H4 and nonhistone protein acetyl-α-tubulin, while α-tubulin as such was taken as the loading control. (**B**) Cells (10^5^/well) were seeded in 6-well plates and allowed to attach overnight. The day after, increasing amounts of (S)-2 (0, 2.5 and 5 μM) were added to the wells and viable cells (trypan blu-negative) were counted with the aid of a Bürker chamber at the indicated time points (results were the mean ± SD of experiments done in quadruplicate). (**C**) Phase contrast pictures of companion cultures showed that (S)-2 induced morphological changes and a marked decrease in cell density (a typical experiment out of three). (**D**) For cell cycle distribution HCT116 cultures were treated without/with 5 μM (S)-2 for 48 h and then incubated with a PI/RNase solution for 30 min at 4°C prior to the flow cytometric analysis. The percentage of cells in the different phases of the cell cycle was calculated by the ModFit program and shown in each panel were reproduced in three separate experiments. (**E**) top – Apoptosis in cell cultures treated without/with the drug was also assessed cytofluorimetrically by using the Annexin-V-Fluos/PI assay and the calculated percentages of four different assays yielded comparable values. (E) bottom – Cell extracts of HCT116 incubated for the indicated time points with 5 μM (S)-2 were subjected to Western immunoblot analysis of the PARP cleaved fragment; GAPDH was the loading control. (**F**) Cell cultures were pre-incubated for 2 h with the pan-caspase inhibitor Z-VAD-fmk (30 μM) and then were treated without/with 5 μM (S)-2 for 24 h. Cell lysates were analyzed by Western immunoblot to detect the cleavage of PARP and of caspase 9; GAPDH was the loading control.

The BDZ-hydroxamate hybrid (S)-2 was assayed for its HDAC-inhibitory activity by using the human colorectal cancer cell line HCT116 as the primary model. Western blot analyses showed that incubation of cultures with 5 μM (S)-2 enhanced histone H3 acetylation and prompted *de novo* acetylation of both H4 and α-tubulin (Figure 1A). These effects were observed as early as at 6 h and remained steady up to 48 h of treatment, and were accompanied by a dose-dependent cell growth arrest (Figure 1B). Moreover, typical HCT116 culture monolayers underwent morphological changes upon incubation with (S)-2 that induced a marked cell detachment from the substrate, while the residual attached cells displayed a fairly enlarged phenotype (Figure 1C). In addition, a 48 h-exposure of HCT116 cultures to 5 μM (S)-2 modified cell cycle progression as indicated by a nearly three-fold increase of cells arrested in G2/M-phase relative to control, and a large decrease of cell population in S-phase (from about 37% of controls to 7.2% of treated cultures) (Figure 1D). Besides, about half of HCT116 treated cells underwent apoptosis as assessed by flow cytometry at 48 h (Figure 1E, top); and consistently, western blots analyses of cell extracts from drug-treated cultures showed that (S)-2 caused the cleavage of caspase substrate poly(ADP-ribose) polymerase (PARP), a well-known pro-apoptotic marker (Figure 1E, bottom). Moreover, to clarify mechanisms of drug-induced apoptosis in HCT116 cells, we used the pan-caspase inhibitor Z-VAD-fmk that was added in culture at 30 μM concentration just 2 h prior to a 24 h-treatment without/with 5 μM (S)-2. The inhibitor abolished (S)-2-mediated activation of caspase cascade and the cleavage of PARP thus indicating that the apoptotic process developed through a caspase-dependent pathway (Figure 1F).

### (S)-2 modulates the GSK-3β/β-catenin signaling pathway

**Figure 2:**
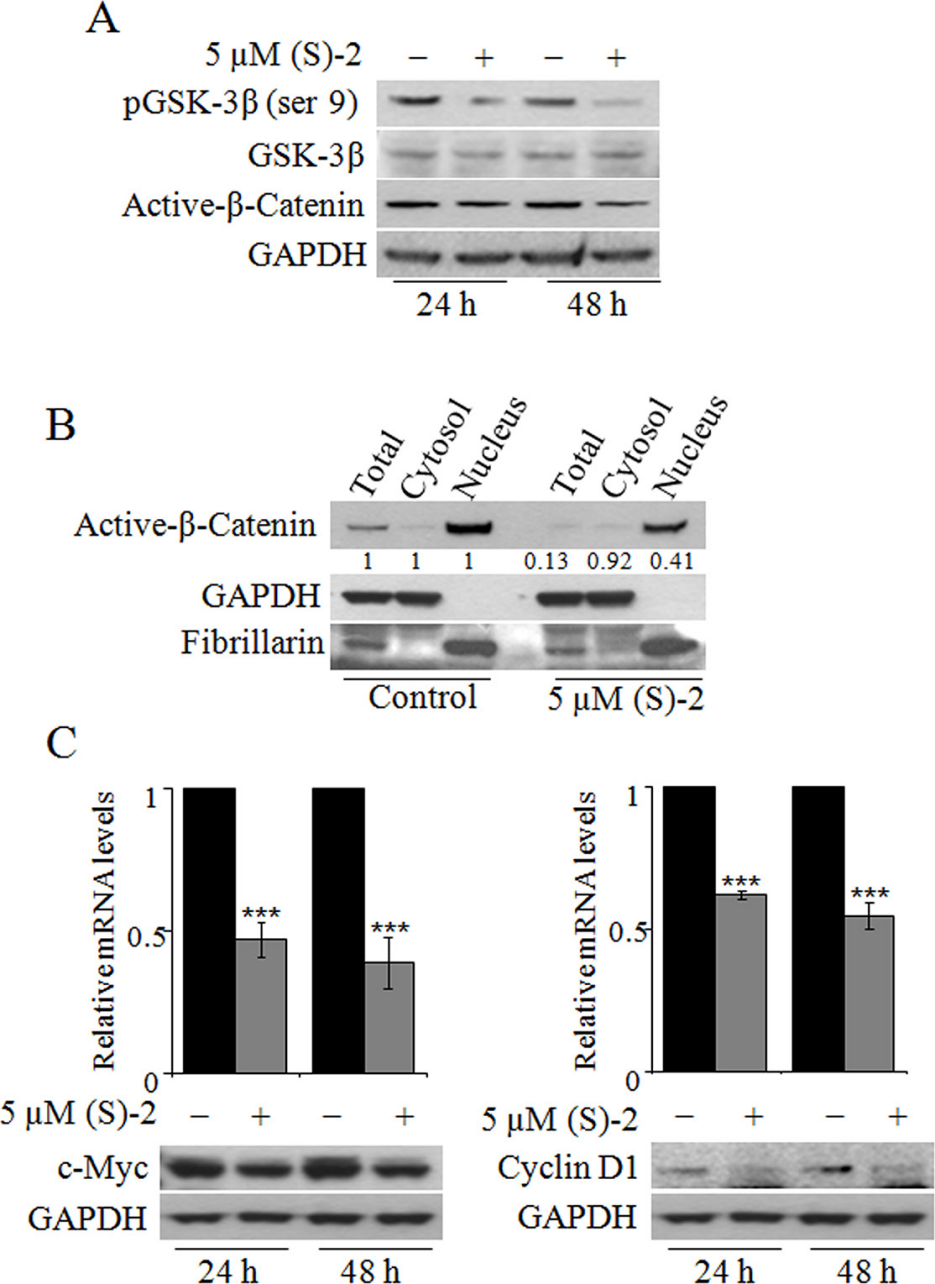
The effects of (S)-2 on GSK-3β/β-catenin pathway (**A**) Cell extracts were analyzed by Western blot to detect phospho-GSK-3β(ser9), GSK-3β and active-β-Catenin levels; GAPDH was used as the loading control. (**B**) The total, cytosolic and nuclear extracts of HCT116 cells treated for 48 h without/with 5 μM (S)-2 were obtained (see Materials and Methods) and probed for the active-β-Catenin levels. Purity of the two subcellular fractions was assessed by the presence of GAPDH and fibrillarin as the markers of the cytosolic and nuclear compartment, respectively. (**C**) mRNA and protein levels of c-Myc and cyclin D1 in HCT116 cells treated without/with 5 μM (S)-2 for 24 h and 48 h were determined by quantitative real-time PCR (***P ≤ 0.01) and Western blot, respectively.

An activating mutation of the Wnt/β-catenin pathway is a key oncogenic event occurring in almost all CRCs [26, 27]. Due to this specific alteration the GSK-3β-mediated degradation of β-catenin in the cytoplasm is inhibited and this allows the protein accumulation of in the nucleus where it acts as a transcription factor [28]. It seemed interesting, therefore, to explore the effectiveness of (S)-2 on GSK-3β/β-catenin signaling pathway in HCT116 cells. Immunoblot analyses of total cell extracts from cultures incubated up to 48 h with 5 μM (S)-2 showed a time-dependent decrease in the levels of both pGSK-3β(ser9) and active-β-catenin (Figure 2A). The evaluation of active β-catenin amounts in total cell lysates as well as in the cytosolic and nuclear fractions after 48 h of treatment proved that the drug caused a significant decrease of total active-β-catenin that, however, was mainly confined to the nucleus (Figure 2B). Furthermore, as *c-Myc* and *cyclin D1* are well-known target oncogenes of β-catenin transcriptional activity [26, 28] we monitored their expression following treatment without/with 5 μM (S)-2 for 24 h and 48 h, and observed a significant drug-induced decrease of mRNA and protein levels (Figure 2C, top and bottom panel, respectively).

**Figure 3:**
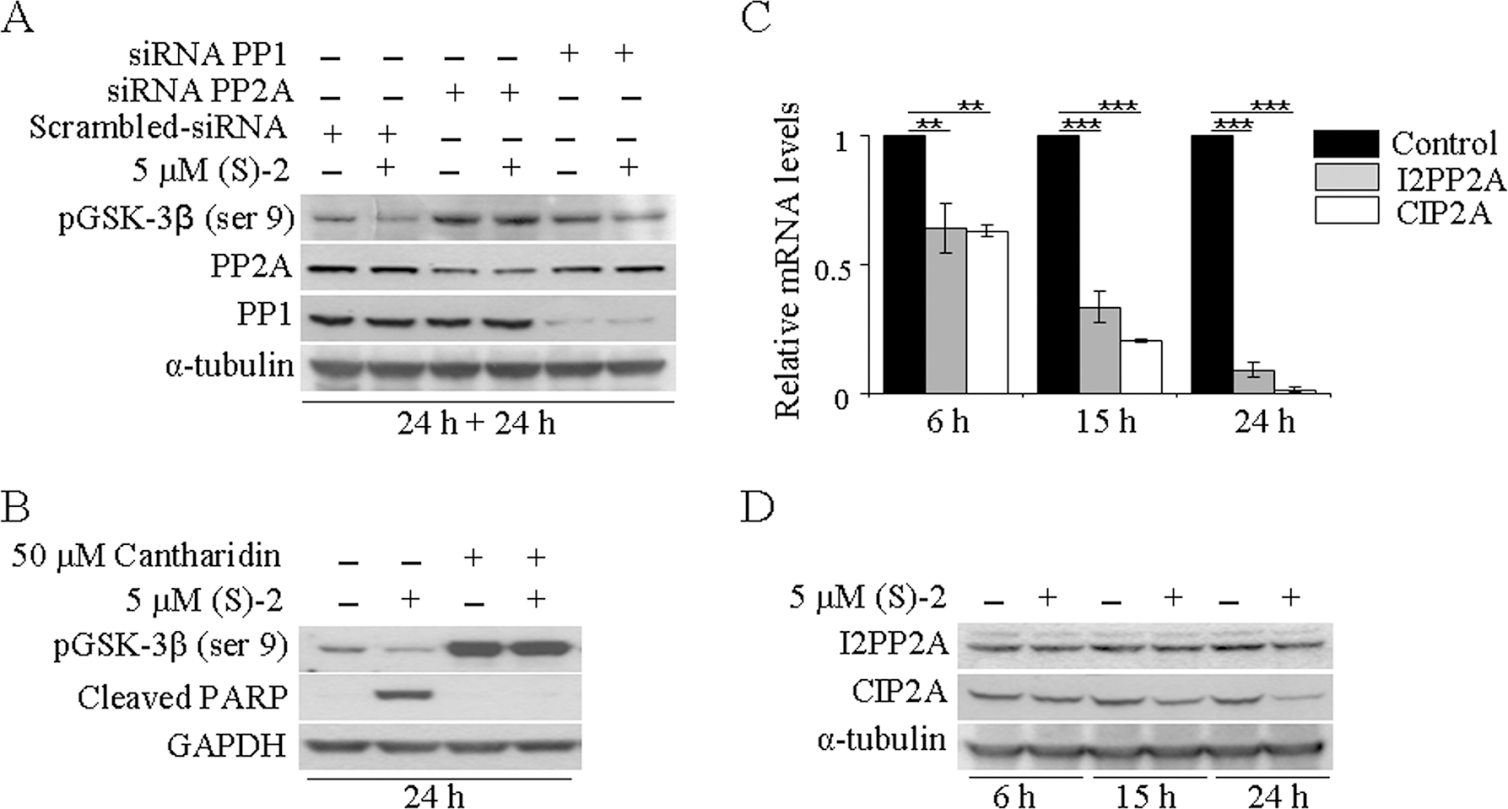
PP2A is responsible for drug-mediated pGSK-3β dephosphorylation (**A**) HCT116 cells transfected with pool-siRNAs towards either *PP1* or *PP2A* for 24 h were incubated for additional 24 h without/with 5 μM (S)-2; then cell extracts were analyzed by Western immunoblot to detect levels of pGSK-3β(ser9), PP1 and PP2A; α-tubulin was used as the loading control. (**B**) HCT116 cell cultures were pre-incubated for 2 h with 50 μM Cantharidin and then treated without/with 5 μM (S)-2 for 24 h. Cell lysates were analyzed by Western blot an probed with specific antibodies against pGSK-3β(ser9) and cleaved PARP fragment; GAPDH was the reference protein. (**C** and **D**) mRNA and protein levels of either I2PP2A and CIP2A from HCT116 cells treated without/with 5 μM (S)-2 were determined at the indicated time points by quantitative real-time PCR (**P ≤ 0.05; ***P ≤ 0.01) and Western blot, respectively.

### PP2A, rather than PP1, plays a role in drug-mediated GSK-3β dephosphorylation

Once determined that (S)-2 induced GSK-3β activation through serine dephosphorylation, it was important to identify which serine/threonine protein phosphatases could actually be involved. As the bulk of cellular serine/threonine phosphatases is represented by PP1 and PP2A phosphatases [29], their roles in drug-mediated pGSK-3β(ser9) dephosphorylation in HCT116 cells have been investigated with the aid of siRNAs. Western blots of lysates of cells transfected with either *PP1* or *PP2A* specific siRNAs for 24 h and then incubated for additional 24 h without/with 5 μM (S)-2 demonstrated that the down-regulation of *PP1* and *PP2A* led to a marked increase in pGSK-3β(ser9), but only the down-regulation of *PP2A* was really effective in abrogating drug-induced dephosphorylation of pGSK-3β(ser9) (Figure 3A). These data were confirmed by experiments carried out with Cantharidin, a chemical inhibitor of PP2A [30], that was added at 50 μM concentration to HCT116 cell cultures 2 h prior to a 24 h-treatment without/with (S)-2. Cantharidin alone prompted a marked increase in pGSK-3β(ser9) and also abolished drug-induced dephosphorylation of pGSK-3β(ser9) and PARP cleavage thus blocking the apoptotic process (Figure 3B). Moreover, further information on mechanisms responsible for (S)-2-mediated activation of PP2A were provided by monitoring the expression of its endogenous cellular inhibitors, *i.e.* the inhibitor 2 of protein phosphatase 2A (I2PP2A) and the cancerous inhibitor of protein phosphatase 2A (CIP2A) [31]. Relative mRNA levels of both *I2PP2A* and *CIP2A* underwent a significant time-dependent decrease upon culture treatment with 5 μM drug (Figure 3C). Conversely, immunoblot analyses showed that while the amount of I2PP2A was steady along with the experiment, the CIP2A signal decreased at 15 h and more considerably at 24 h thus suggesting that, indeed, this was the phosphatase inhibitor playing a major role in (S)-2-mediated PP2A activation (Figure 3D).

### (S)-2-induced downregulation of CIP2A is mediated by the inhibition of HDAC1

**Figure 4:**
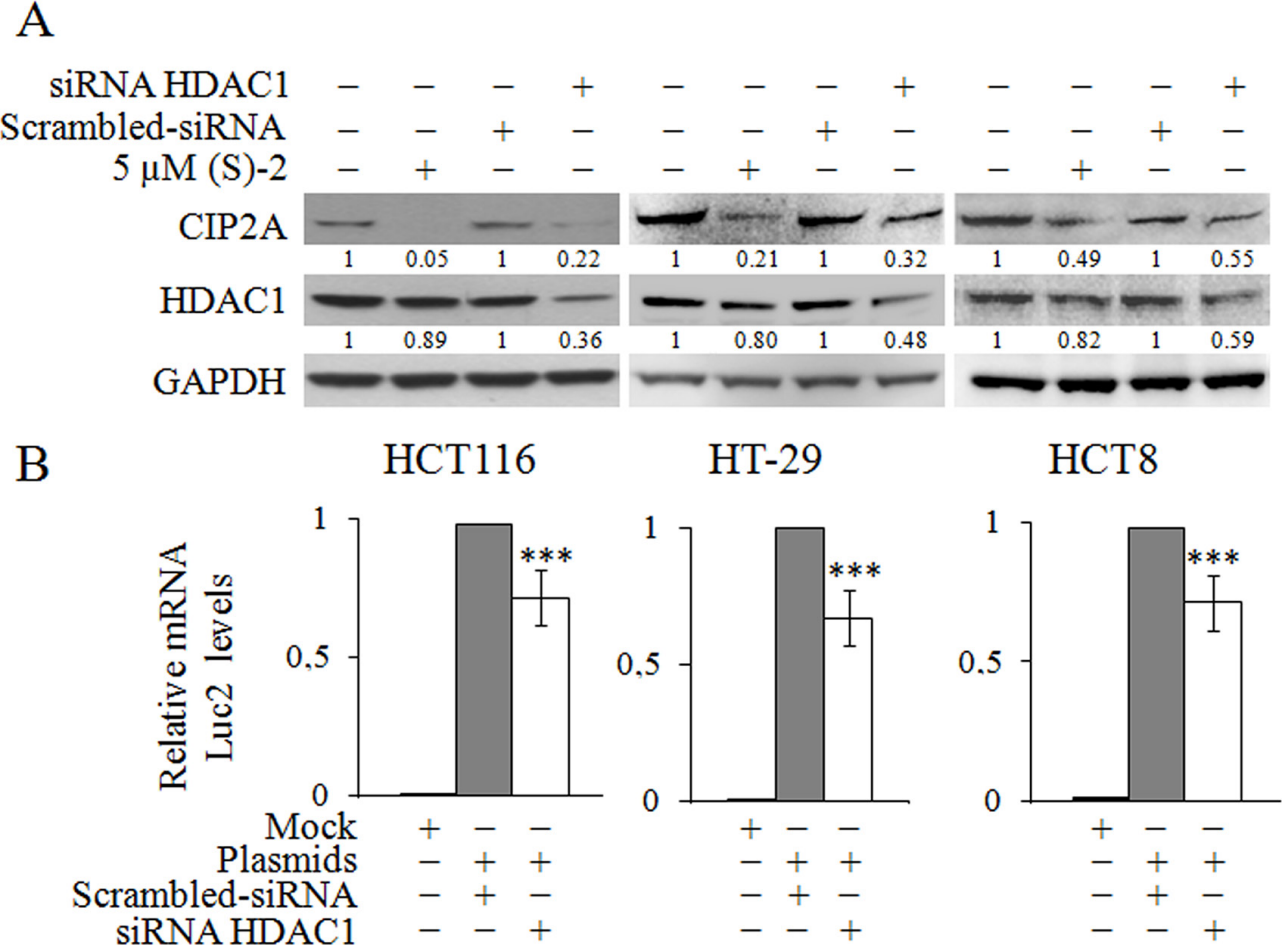
Inhibition of HDAC1 induces CIP2A downregulation (**A**) HCT116, HT-29 and HCT8 cell cultures were treated without/with 5 μM (S)-2 for 24 h or transfected with either *HDAC1*-specific and scrambled siRNAs for 48 h. Cell lysates were submitted to Western immunoblot to detect HDAC1 and CIP2A levels; GAPDH was used as the control protein. (**B**) Cells were first treated with *HDAC1*-specific siRNA and scrambled siRNA for 48 h, and then co-transfected with either 1082CIP2ALuc-pGL4.10 or pGL4.10 plasmids and with pGL4.70 up to 16 h. *Luc2* and *Renilla* mRNA levels were determined by quantitative real-time PCR (**P ≤ 0.05; ***P ≤ 0.01).

The drug-mediated decrease of *CIP2A* expression in HCT116 cells has clearly indicated that HDACs were involved in the regulation of this particular gene; and, therefore, we evaluated the effects of specific siRNAs towards the nuclear histone deacetylase *HDAC1* that we previously reported to be a sensitive target of (S)-2 [21]. Western blot analyses showed that HCT116 cells treated without/with either 5 μM (S)-2 and/or specific *HDAC1* siRNA led to a decrease in CIP2A protein signal (Figure 4A), hence suggesting that the drug-induced decrease of CIP2A could actually develop through the inhibition of HDAC1. And, notably, similar changes have also been reproduced in different CRC cell lines such as HT-29 and HCT8. The direct role of HDAC1 in governing CIP2A expression in CRC cells has further been confirmed with the aid of a plasmid harboring the human *CIP2A* full length promoter fused with the *Luc2* luciferase gene. In fact, the downregulation of HDAC1 protein signal by specific siRNA led to a significant decrease of mRNA levels of *Luc2* (Figure 4B) due to the silencing the *CIP2A* promoter in all the three CRC cell lines.

### (S)-2 greatly reduces HCT116 cell proliferation in mice xenografts

**Figure 5:**
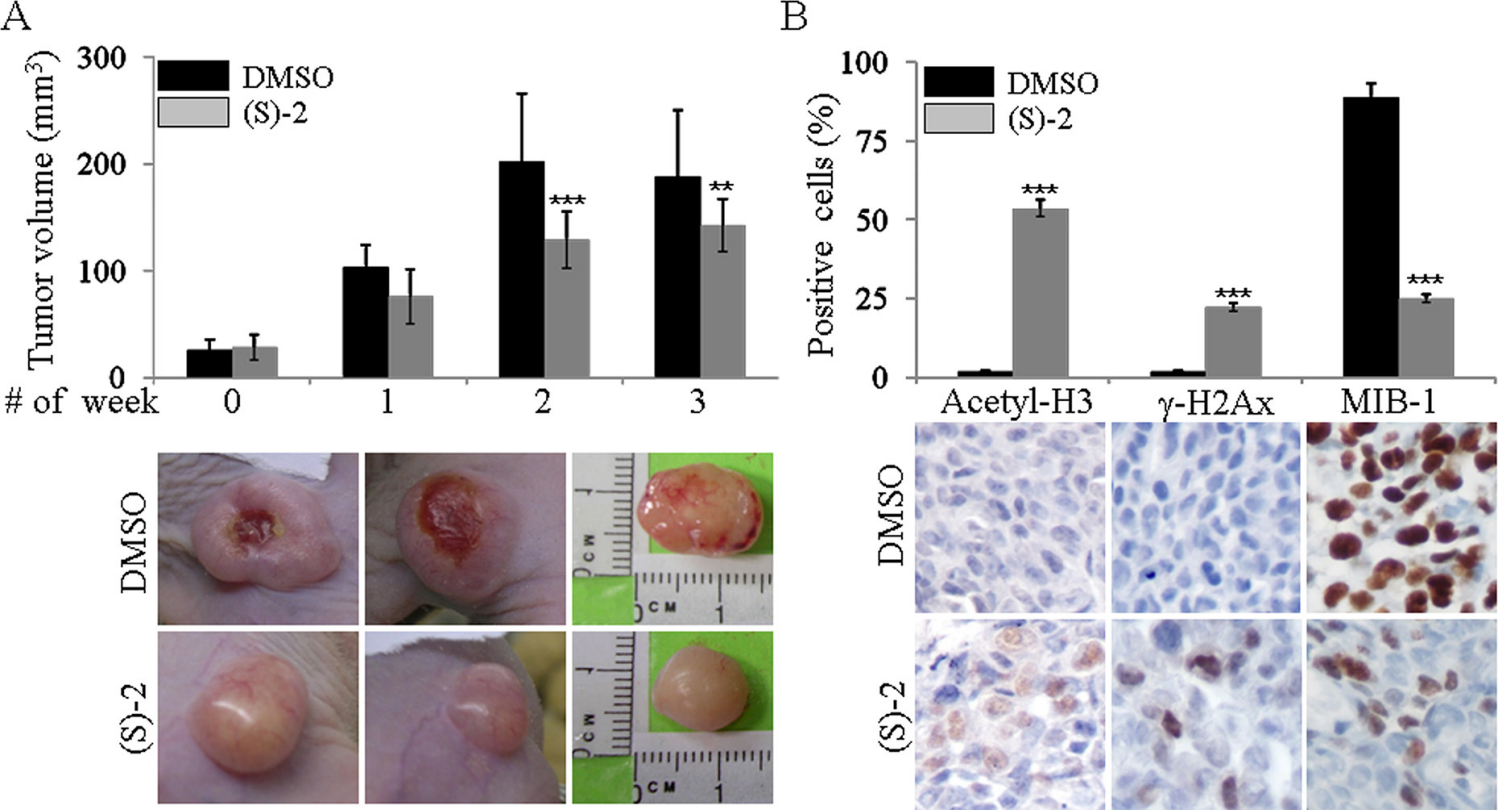
Tumour xenograft (**A**) top – Aliquots of HCT116 cell suspension (2.5 × 10^6^ cell/100 μl RPMI) were injected subcutaneously in both flanks of male nude mice (see also as Materials and Methods). Positively-xenografted mice were randomized into two groups and then injected *ip* with either the drug or DMSO as the vehicle. Treatments were administered three times a week for the first two weeks and twice in a row on the last week when mice were sacrificed by cervical dislocation. Variations in the tumour volumes along with the experiment and after the sacrifice were measured by a caliper. (A) bottom – Excised tumours were weighed and volumes were calculated according to the formula (length (mm)) × (width (mm)) × (depth (mm)) × p/6. Statistical analyses of changes in tumour volumes (mean ± SD) after 0, 3, 6 and 8 treatments were as follows: [for untreated mice: #0 (25.7 ± 10.0); #3 (103.3 ± 20.6); #6 (201.7 ± 64.2); #8 (188.6 ± 62.1)] and [for treated mice: #0 (28.2 ± 11.7); #3 (76.1 ± 25.5); #6 (128.6 ± 26.5); #8 (142.2 ± 24.7)]. According to these values the drug was capable of reducing tumour volumes of about 26.4% (1st week), 36.2% (2nd week) and 24.6% (3rd week), as compared to control. Photographs are representative of tumour masses from mice treated with either the vehicle or (S)-2, respectively. (**B**) top – Immunohistochemistry was performed on specimens of human colon cancer xenografts by using primary antibodies against acetyl-H3 and γ-H2AX, and also the monoclonal antibody MB-1 that recognizes the nuclear marker Ki-67 associated to cell proliferation (see Materials and Methods) followed by a peroxidase-conjugated IgG preparation; 3,3′-diaminobenzidine was employed as the chromogen for development. Slides were counterstained with aqueous Meyer hematoxylin and mounted with glycerol for visual inspection and photography. (B) bottom – Statistical analyses of data on both top panels of the figure were carried out by Student's *t*-test and significant differences between the two groups were indicated by the asterisks (**P < 0.05; ***P < 0.01).

To assess the *in vivo* anticancer effectiveness of (S)-2 in a xenograft tumor model, HCT116 cells were implanted onto both flanks of nude mice (see legend to Figure 5 and Materials and Methods for details). A week later, a small swelling was perceived under the skin of some of the animals which were assumed as positively-xenografted mice which were randomized into two groups and then injected *ip* with either the drug or the vehicle (DMSO). Treatments were administered three times a week for the first two weeks and twice in a row on the last week when mice were sacrificed by cervical dislocation. Changes in tumour volumes during along with the experiment were monitored by measurements with a caliper. Tumour mass volumes of (S)-2-treated mice were significantly smaller (average ± 30%) than those of untreated mice (Figure 5A, top). Besides, tumour masses of untreated mice were hyperemic and underwent ulceration, whereas those of drug-treated mice did not burst and remained fairly pale in color to suggest that tumour growth elicited only a modest angiogenesis (Figure 5A, bottom).

Immunohistochemistry of tumour specimens was performed to determine *in vivo* effects of (S)-2 (Figure 5B). We found that the drug exerted its activity within the tumour cells as shown by (a) the presence of acetyl-H3 and γ-H2AX (the latter denoting drug-induced caspase activation and DNA damages) and (b) the marked decrease, as compared to untreated mice, in the amount of tumour cells positively-stained with MIB-1 (against the nuclear marker Ki-67 associated with cell proliferation [32–34].

## DISCUSSION

**Figure 6:**
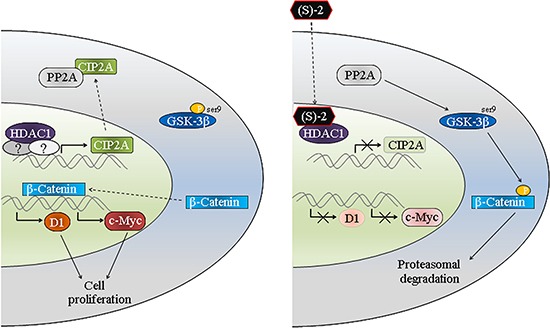
Schematic diagram of (S)-2-induced growth arrest and apoptosis in colon cancer cells The oncogenic protein HDAC1 acts as a transcription factor for CIP2A that afterward complexes and inactivates PP2A. The latter becomes unable to dephosphorylate pGSK-3β(ser9) thus allowing the translocation of β-catenin in the nucleus where it induces the transcription of *c-Myc* and *cyclin D1* and, consequently, cell proliferation. The inhibition of HDAC1 by (S)-2 leads to the downregulation of CIP2A expression and, thus, restores the activity of PP2A that dephosphorylates pGSK-3β(ser9). The activated kinase GSK-3β phosphorylates β-catenin that undergoes degradation by the ubiquitin-proteasome system. The decrease in β-catenin levels downregulates *c-Myc* and *cyclin D1* expression and, consistent with this, leads to cell growth arrest.

The anticancer properties of (S)-2 towards CRC cells have been thoroughly described in the previous section. Indeed, the drug induced cell cycle arrest and caspase-dependent apoptosis in HCT116 cells and also proved effective *in vivo* by contrasting tumour growth in mice xenografts. However, moving beyond a plain list of all biological and molecular events characterizing the anticancer activity of (S)-2 in CRC cells, we would focus on a specific result that emerged during this study. HDAC1 is an oncogenic protein acting as a transcription factor of various genes [35] among which, however, *CIP2A* was not included previously. *CIP2A* is also an oncoprotein overexpressed in several cancers including CRCs [25, 36] where it inhibits the activity of oncosuppressor PP2A that controls cell cycle and apoptosis [36–39]. PP2A inhibition suppresses the dephosphorylation of pGSK-3β(ser9), thus inhibiting β-catenin degradation and then maintaining the activity of canonical Wnt/β-catenin pathway. Results herein (see the schematic diagram, Figure 6) show that the inhibition of HDAC1, by either (S)-2 or specific siRNA, downregulates CIP2A and, therefore, restores the activity of PP2A that dephosphorylates pGSK-3β(ser9). Upon activation, GSK-3β phosphorylates β-catenin that remains confined to the cytosol where it undergoes degradation. The decreased amount/activity of the β-catenin transcription factor induces cell growth arrest by diminishing *c-Myc* and *cyclin D1* expression and abrogating the prosurvival Wnt/β-catenin signaling pathway as well. This is the first time that a direct effect of HDAC1 on *CIP2A* transcription is reported to occur in HCT116, HT-29 and HCT8, thus inferring that this molecular link might be an original signature shared by various types of CRC cells. In this context, the HDACi (S)-2 represented the tool capable of unleashing PP2A activity by inducing HDAC1-mediated downregulation of *CIP2A* transcription in human colon cancers.

## MATERIALS AND METHODS

### Cell culture and treatments

Human colon cancer cells HCT116 (CCL-247, ATCC), HT-29 (HTB-38, ATCC) and HCT8 (CCL-244, ATCC), have been cultured in RPMI 1640 supplemented with 10% fetal bovine serum (FBS, EuroClone, Life Science Division, Milan, Italy) and 2 mM L-glutamine at 37°C in 5% CO_2_ humidified atmosphere. (S)-2 was dissolved in dimethyl sulfoxide (DMSO; Sigma-Aldrich, ST. Louis, MO, USA) at 0.1 M concentration and stored in the dark at room temperature (RT). Working drug solutions were obtained by appropriate dilution of the stock solution with the culture medium. The amount of DMSO employed as vehicle did not interfere with drug biological activities. In caspase inhibition experiments Z-VAD-fmk (R&D Systems, Minneapolis, MN, USA) was added in culture 2 h prior to treatment with (S)-2. Cantharidin (Santa Cruz Biotechnology, Santa Cruz, CA, USA) used to inhibit protein phosphatase 2A (PP2A) activity was dissolved in DMSO at 0.1 mM final concentration and stored in −20°C; the inhibitor, at the established concentrations, was delivered to cultures 2 h prior to treatment.

### Cell cycle analysis and determination of apoptosis

Cell cycle phases were assessed by the propidium iodide (PI)-hypotonic citrate method; apoptosis was measured by the Annexin-V-Fluos/PI test (Roche Molecular Biochemicals, Mannheim, Germany) with the aid of Becton Dickinson FACSCalibur System (Becton-Dickinson, San Jose, CA, USA) [21].

### Western blotting

Harvested cells were resuspended in 20 mM RIPA buffer (pH 7.4) containing a cocktail of proteinase inhibitors (Calbiochem, Merck, Darmstadt, Germany) and treated by sonication (Microson XL-2000, Minisonix, Farmingdale, NY, USA) to obtain a total protein extract. For cytosol-nucleus fractionation, proteins were isolated by using the ProteoExtract Subcellular Proteome Extraction Kit (Calbiochem) as suggested by the manufacturer. Proteins were assayed by the BCA Protein Assay (Thermo Scientific, Rockford, IL, USA), analyzed by SDS-PAGE and western blotting as reported elsewhere [40]. Membranes were probed with primary antibodies against acetyl-H3, acetyl-H4, active-β-catenin, and PP2A (Upstate Biotechnology, Millipore, Bilerica, MA, USA); acetyl-α-tubulin and α-tubulin (Sigma-Aldrich); GAPDH, cleaved PARP, cleaved caspase-9, c-Myc and phospho-GSK-3β(ser9) (Cell Signaling Technology, Danvers, MA. USA); cyclin D1, GSK-3β, I2PP2A, CIP2A and fibrillarin (Santa Cruz Biotechnology); suitable peroxidase-conjugated IgG preparations (Sigma-Aldrich) were used as secondary antibodies; the ECL procedure was employed for development. Western immunoblots reported all through this work were the mean ± SD of experiments carried out in triplicate (unless otherwise specified) and the software ImageJ [41] was used for densitometric quantification of protein band intensity.

### Quantitative real time-PCR analysis

QRT-PCR was performed with reverse transcripted cDNA of either untreated or drug-treated cells by using the Applied Biosystems 7500HT System according to standard protocols. Fold of *c-Myc*, *cyclin D1*, *I2PP2A* and *CIP2A* induction were calculated by the changes of each of their Ct values in treated *vs.* untreated cells and were normalized to the *18S rRNA* Ct values. Amplification was performed with the default PCR setting: 40 cycles of 95°C for 15 sec and of 60°C for 60 sec using a SYBR Green based detection (SYBR Green Master mix; Applied Biosystems, Thermo Fisher Scientific, Waltham, MA, USA) and the following primers: *cyclin D1* forward 5′-CGTGGCCTCTAAGATGAAGG-3′ and reverse 5′-GTGTTCAATGAAATCGTGCGG-3′; *c-Myc* forward 5′-TCAAGAGGTGCCACGTCTCC-3′ and reverse 5′-TCTTGGCAGGATAGTCCTT-3′; *I2PP2A* forward 5′-CGTTCGAGTCAAACGCAGAA-3′ and reverse 5′-CAGCACCTGCATCAGAATGGT-3′; *CIP2A* forward 5′-TGACCCTTCTGCTGCCTACA-3′ and reverse 5′-GCCTTGGCAATCCTTTCACA-3′; *18S rRNA* forward 5′-CGGCTACCACATCAAGGAA-3′ and reverse 5′-GCTGGAATTACCGCGGCT-3.′

### siRNAs and plasmids transfection

In silencing experiments, 2 × 10^5^ cells were seeded in 60 mm culture dishes 16 h before transfection with siRNAs using 7.5 μl of Lipofectamine RNAiMAX (Invitrogen, Thermo Fisher Scientific). *PP1* (mix of 500 pmol of each #105828 targeting *PPP1CA*, #104504 targeting *PPP1CB* and #105829 targeting *PPP1CC*), *PP2A* (mix of 500 pmol of each #104510 targeting *PPP2CA* and #104717 targeting *PPP2CB*), *HDCA1* (500 pmol of #120418) and non-targeting control siRNA (500 pmol of #4390844) were from Thermo Fisher Scientific. In over-expression experiments, 2 × 10^5^ cells were seeded into 60 mm dishes 16 h before co-transfection with 2.5 μg of plasmid 1082CIP2ALuc-pGL4.10 (kind gift of Professor Jukka Westermark, University of Turku and Abo Akademi, Turku, Finland) [42] or pGL4.10 (as control, Promega, Fitchburg, WI, USA) and 2.5 μg pGL4.70 (Promega) using 7.5 μl of Lipofectamine LTX (Invitrogen, Thermo Fisher Scientific).

### Tumour xenograft model

Male nude (nu/nu) mice (Harlan Laboratories, Srl, San Pietro al Natisone, UD, Italy) were cared for and maintained in accordance with applicable European Animal Welfare regulations under an approved Institutional Animal Care and Use Protocol in an animal facility at University of Florence accredited by the Association for Assessment and Accreditation of Laboratory Animal Care. To establish subcutaneous tumours, aliquots of HCT116 cell suspension (2.5 × 10^6^ cell/100 μl RPMI) were injected subcutaneously on both flanks of 24 mice. A week later the tumour mass was perceived in 14 mice (out of those originally injected), which were then randomized into two equal groups and treated with the drug or the vehicle at the established times. Changes in tumour volumes during the experiment were monitored by regular measurements with a caliper. Eventually, mice were killed by cervical dislocation and tumours were excised, weighed and their volumes were calculated according to the formula (length (mm)) × (width (mm)) × (depth (mm)) × p/6.

### Immunohistochemistry

Slides with consecutive 2.5–5 μm sections of paraffin embedded tumour masses were first deparaffinized, boiled in 1 mM EDTA pH 9 for 15 min and after cooling aspecific peroxidases were blocked with 3% H_2_O_2_ for 10 min. Then, slides were treated according to standard procedures and incubated with primary antibody solutions against acetyl-H3 and γ-H2AX (to denote drug-induced caspase activation and DNA damages); and also with the monoclonal antibody MIB-1 (recognizing the nuclear marker Ki-67 that was associated with cell proliferation) (Dako, Glostrup, Danimarca) [33, 34] followed by a peroxidase-conjugated IgG preparation; 3,3′-diaminobenzidine (Zymed Laboratories Inc., South San Francisco, CA, USA) was used as the chromogen for development. Slides were counterstained with aqueous Meyer hematoxylin and mounted with glycerol for visual inspection and photography; pictures are representative of four randomly chosen microscopic fields (magnification: ×400) and taken with the aid of a microscope (Nikon Eclipse, mod. 50i) equipped with a digital camera (DS-5 M USB2) (Nikon Instruments, Florence, Italy) as described previously [22].

### Statistical analysis

The Student′s *t*-test or one-way analysis of variance have been employed to assess statistical significance of results. The difference among the values was considered significant at *P* ≤ 0.05.

## References

[1] Strahl BD, Allis CD (2000). The language of covalent histone modifications. Nature.

[2] Grunstein M (1997). Histone acetylation in chromatin structure and transcription. Nature.

[3] Struhl K (1998). Histone acetylation and transcriptional regulatory mechanisms. Genes Dev.

[4] Narlikar GJ, Fan HY, Kingston RE (2002). Cooperation between complexes that regulate chromatin structure and transcription. Cell.

[5] (2009). Beware of neuronal Sunday drivers: Sunday Driver interacts with two distinct classes of axonal organelles. J Biol Chem.

[6] Marks P, Rifkind RA, Richon VM, Breslow R, Miller T, Kelly WK (2001). Histone deacetylases and cancer: causes and therapies. Nat Rev Cancer.

[7] Kouzarides T (1999). Histone acetylases and deacetylases in cell proliferation. Curr Opin Genet Dev.

[8] Monneret C (2005). Histone deacetylase inhibitors. Eur J Med Chem.

[9] Bolden JE, Peart MJ, Johnstone RW (2006). Anticancer activities of histone deacetylase inhibitors. Nat Rev Drug Discov.

[10] Marks PA, Xu WS (2009). Histone deacetylase inhibitors: Potential in cancer therapy. J Cell Biochem.

[11] Mai A, Altucci L (2009). Epi-drugs to fight cancer: from chemistry to cancer treatment, the road ahead. Int J Biochem Cell Biol.

[12] Johnstone RW (2002). Histone-deacetylase inhibitors: novel drugs for the treatment of cancer. Nat Rev Drug Discov.

[13] Glozak MA, Seto E (2007). Histone deacetylases and cancer. Oncogene.

[14] Chen CS, Weng SC, Tseng PH, Lin HP (2005). Histone acetylation-independent effect of histone deacetylase inhibitors on Akt through the reshuffling of protein phosphatase 1 complexes. J Biol Chem.

[15] Balliu M, Guandalini L, Romanelli MN, D'Amico M, Paoletti F (2015). HDAC-inhibitor (S)-8 disrupts HDAC6-PP1 complex prompting A375 melanoma cell growth arrest and apoptosis. J Cell Mol Med.

[16] Glozak MA, Sengupta N, Zhang X, Seto E (2005). Acetylation and deacetylation of non-histone proteins. Gene.

[17] Bode AM, Dong Z (2004). Post-translational modification of p53 in tumorigenesis. Nat Rev Cancer.

[18] Chen L, Fischle W, Verdin E, Greene WC (2001). Duration of nuclear NF-kappaB action regulated by reversible acetylation. Science.

[19] Roy S, Packman K, Jeffrey R, Tenniswood M (2005). Histone deacetylase inhibitors differentially stabilize acetylated p53 and induce cell cycle arrest or apoptosis in prostate cancer cells. Cell Death Differ.

[20] Guandalini L, Cellai C, Laurenzana A, Scapecchi S, Paoletti F, Romanelli MN (2008). Design, synthesis and preliminary biological evaluation of new hydroxamate histone deacetylase inhibitors as potential antileukemic agents. Bioorg Med Chem Lett.

[21] Cellai C, Balliu M, Laurenzana A, Guandalini L, Matucci R, Miniati D, Torre E, Nebbioso A, Carafa V, Altucci L, Romanelli MN, Paoletti F The new low-toxic histone deacetylase inhibitor S-(2) induces apoptosis in various acute myeloid leukaemia cells. J Cell Mol Med.

[22] Laurenzana A, Balliu M, Cellai C, Romanelli MN, Paoletti F Effectiveness of the histone deacetylase inhibitor (S)-2 against LNCaP and PC3 human prostate cancer cells. PloS one.

[23] Khanna A, Pimanda JE, Westermarck J (2013). Cancerous inhibitor of protein phosphatase 2A, an emerging human oncoprotein and a potential cancer therapy target. Cancer Res.

[24] De P, Carlson J, Leyland-Jones B, Dey N (2014). Oncogenic nexus of cancerous inhibitor of protein phosphatase 2A (CIP2A): an oncoprotein with many hands. Oncotarget.

[25] Wiegering A, Pfann C, Uthe FW, Otto C, Rycak L, Mader U, Gasser M, Waaga-Gasser AM, Eilers M, Germer CT (2013). CIP2A influences survival in colon cancer and is critical for maintaining Myc expression. PloS one.

[26] Bos CL, Kodach LL, van den Brink GR, Diks SH, van Santen MM, Richel DJ, Peppelenbosch MP, Hardwick JC (2006). Effect of aspirin on the Wnt/beta-catenin pathway is mediated via protein phosphatase 2A. Oncogene.

[27] Serafino A, Moroni N, Zonfrillo M, Andreola F, Mercuri L, Nicotera G, Nunziata J, Ricci R, Antinori A, Rasi G, Pierimarchi P (2014). WNT-pathway components as predictive markers useful for diagnosis, prevention and therapy in inflammatory bowel disease and sporadic colorectal cancer. Oncotarget.

[28] McCubrey JA, Steelman LS, Bertrand FE, Davis NM, Abrams SL, Montalto G, D'Assoro AB, Libra M, Nicoletti F, Maestro R, Basecke J, Cocco L, Cervello M (2014). Multifaceted roles of GSK-3 and Wnt/beta-catenin in hematopoiesis and leukemogenesis: opportunities for therapeutic intervention. Leukemia.

[29] Brush MH, Guardiola A, Connor JH, Yao TP, Shenolikar S (2004). Deactylase inhibitors disrupt cellular complexes containing protein phosphatases and deacetylases. J Biol Chem.

[30] Honkanen RE (1993). Cantharidin, another natural toxin that inhibits the activity of serine/threonine protein phosphatases types 1 and 2A. FEBS letters.

[31] Ciccone M, Calin GA, Perrotti D (2015). From the Biology of PP2A to the PADs for Therapy of Hematologic Malignancies. Frontiers in oncology.

[32] Li LT, Jiang G, Chen Q, Zheng JN (2015). Ki67 is a promising molecular target in the diagnosis of cancer (review). Molecular medicine reports.

[33] Gerdes J, Schwab U, Lemke H, Stein H (1983). Production of a mouse monoclonal antibody reactive with a human nuclear antigen associated with cell proliferation. Int J Cancer.

[34] Bullwinkel J, Baron-Luhr B, Ludemann A, Wohlenberg C, Gerdes J, Scholzen T (2006). Ki-67 protein is associated with ribosomal RNA transcription in quiescent and proliferating cells. J Cell Physiol.

[35] Santoro F, Botrugno OA, Dal Zuffo R, Pallavicini I, Matthews GM, Cluse L, Barozzi I, Senese S, Fornasari L, Moretti S, Altucci L, Pelicci PG, Chiocca S (2013). A dual role for Hdac1: oncosuppressor in tumorigenesis, oncogene in tumor maintenance. Blood.

[36] Khanna A, Pimanda JE (2015). Clinical significance of Cancerous Inhibitor of Protein Phosphatase 2A (CIP2A) in human cancers. Int J Cancer.

[37] Perrotti D, Neviani P (2013). Protein phosphatase 2A: a target for anticancer therapy. The Lancet Oncology.

[38] Eichhorn PJ, Creyghton MP, Bernards R (2009). Protein phosphatase 2A regulatory subunits and cancer. Biochimica et biophysica acta.

[39] Perrotti D, Neviani P (2008). Protein phosphatase 2A (PP2A), a drugable tumor suppressor in Ph1(+) leukemias. Cancer metastasis reviews.

[40] Laurenzana A, Cellai C, Vannucchi AM, Pancrazzi A, Romanelli MN, Paoletti F (2005). WEB-2086 and WEB-2170 trigger apoptosis in both ATRA-sensitive and -resistant promyelocytic leukemia cells and greatly enhance ATRA differentiation potential. Leukemia.

[41] Schneider CA, Rasband WS, Eliceiri KW (2012). NIH Image to ImageJ: 25 years of image analysis. Nature methods.

[42] Khanna A, Okkeri J, Bilgen T, Tiirikka T, Vihinen M, Visakorpi T, Westermarck J (2011). ETS1 mediates MEK1/2-dependent overexpression of cancerous inhibitor of protein phosphatase 2A (CIP2A) in human cancer cells. PloS one.

